# Replantation

**Published:** 1872-08

**Authors:** 


*Replantation.*—At a recent meeting of the Odontological Society of England, Mr. Steele stated that he lately had a tooth of his own extracted and reinserted under the following circumstances: The tooth had been for some time in an exquisitely sensitive condition from exposure of the pulp, and gave pain on the least change of temperature. Under the influence of nitrous oxide gas, the tooth was carefully pulled out, so as to prevent straining or tearing of the gum; the dental canal was cleansed, the carious part scraped from the crown, stopping applied in the usual way, and the tooth was replaced in its socket. The operation lasted half an hour. For three or four hours there was a dull, aching pain, which, however, entirely ceased before noon of the following day, though some tenderness remained. This, in turn, disappeared; and by the end of a fortnight the replanted tooth did its duty without any difficulty.

